# Exploring a Facilitator‐Enabled Flexible iSupport for Dementia Program: An Internal Pilot Mixed‐Methods Study

**DOI:** 10.1002/nop2.70774

**Published:** 2026-08-23

**Authors:** Ying Yu, Lily Xiao, Shahid Ullah, Claudia Meyer, Michael Chapman, Kai Ping Tan, Langduo Chen, Julie Ratcliffe, Alison Kitson, Andre Q. Andrade, Craig Whitehead, Sue McKechnie

**Affiliations:** ^1^ School of Nursing and Midwifery, College of Health Adelaide University Adelaide Australia; ^2^ College of Health and Enablement Flinders University Bedford Park South Australia Australia; ^3^ Caring Future Institutes Flinders University Bedford Park South Australia Australia; ^4^ College of Medicine and Public Health Flinders University Bedford Park South Australia Australia; ^5^ Bolton Clarke Research Institute Melbourne Victoria Australia; ^6^ Rehabilitation, Ageing and Independent Living Research Centre Monash University Melbourne Victoria Australia; ^7^ Australian National University Canberra Australian Capital Territory Australia; ^8^ Palliative Care Research Unit, Clare Holland House Canberra Health Services Garran Australian Capital Territory Australia; ^9^ Southern Adelaide Local Health Network Bedford Park South Australia Australia; ^10^ The International Learning Collaborative (ILC) Adelaide Australia; ^11^ Australian Centre for Health Services Innovation (AusHSI) Kelvin Grove Queensland Australia; ^12^ School of Nursing and Midwifery University of Sydney Sydney New South Wales Australia; ^13^ Quality Use of Medicines and Pharmacy Research Centre Adelaide University Adelaide Australia; ^14^ Community Services Resthaven Incorporated Wayville South Australia Australia

## Abstract

**Aims:**

To assess the feasibility, fidelity and preliminary impact of a facilitator‐enabled flexible iSupport for dementia program for family carers of people with dementia in a 6‐month intervention period.

**Design:**

This study was a multi‐centre, mixed‐methods, parallel‐group, internal, block‐pilot, randomised controlled trial with an embedded qualitative descriptive study.

**Methods:**

The study was conducted across two community aged care services and two tertiary hospitals in Australia. Participants were recruited between June 2022 and April 2023. Quantitative data were collected via surveys at baseline and 6 months. Qualitative data were obtained from carer support group meetings and interviews.

**Results:**

Eighty‐four carers were enrolled. The recruitment rate was 10% of eligible carers who were approached, and retention in the intervention group at 6 months was 70%. Findings indicate that carers engaged with the program, although engagement varied across components including use of the iSupport manual, participation in online peer support meetings and interaction via WhatsApp. Preliminary analyses suggested a possible trend toward lower behavioural problems among people living with dementia and lower carer distress responses to these behaviours in the intervention group. However, there was no clear evidence of effects on carers' quality of life, perceived social support, self‐efficacy, or the quality of life of people living with dementia. Findings from the pilot informed modifications to enhance the feasibility of the main trial.

**Conclusion:**

This internal pilot study demonstrated the feasibility of a facilitator‐enabled iSupport program and provided preliminary estimates suggesting potential benefits for behavioural outcomes. The results informed refinements to the main trial and highlight the importance of flexible, facilitator‐supported interventions for carers of people living with dementia. Further research in a fully powered trial is required to confirm effectiveness.

**Implications for the Profession and Patient Care:**

Carers of people living with dementia in the community valued the flexible support provided by iSupport facilitators. This support can be delivered in multiple formats, including online meetings, WhatsApp groups and tailored one‐to‐one facilitator support, allowing flexibility to meet diverse caregiver needs. Such an approach is particularly relevant for nurse‐led multidisciplinary services and settings that provide remote care. In addition, this study offers practical insights into feasibility considerations, such as recruitment, engagement and retention, that may inform the design and implementation of future nursing and community‐based interventions.

**Impact:**

- This study provides preliminary evidence on how facilitator‐enabled flexible support can be implemented in community settings, informing the design and delivery of future dementia care interventions.
- Findings suggest that carers can engage with and benefit from structured, facilitator‐supported interventions delivered in flexible formats and may experience potential benefits that warrant further evaluation.
- Facilitator‐enabled flexible support has the potential to complement existing services for carers of people living with dementia and may help address unmet support needs in community settings.

**Reporting Method:**

We adhered to the CONSORT 2010 statement and its extension for randomised pilot and feasibility trials. We also reported in accordance with the Good Reporting of A Mixed Methods Study (GRAMMS).

**Patient or Public Contribution:**

Participants in this study included carers of people living with dementia in the community, supported by health and social care professionals from the four participating organisations.

**Trial Registration:**

The randomised controlled trial protocol was registered with the Australian and New Zealand Clinical Trials Registry (Registration No. ACTRN12622000199718)

## 1 Introduction

ISupport for Dementia is an internet‐ and hardcopy‐based psychoeducation program, developed by the World Health Organisation (WHO) to support the international community in achieving the Global Action Plan for supporting caregivers of people living with dementia (PLWD) (WHO 2017). This program was adapted for the Australian context and comprises six modules and 30 units to support carers of PLWD in developing the knowledge and skills necessary to care for them at home (Xiao et al. 2021). The Australia iSupport for Dementia program was integrated with facilitator‐enabled carer peer support and needs‐based access to care services to meet carers' expectations for one‐stop‐shop care services. This internal pilot randomised controlled trial (RCT) reports part of the large project entitled ‘Creating a partnership in the iSupport program’ to optimise carers' impact on dementia care, which includes three phases. Phase 1 explored the facilitator's role and responsibilities. Phase 2 was an RCT to test the effectiveness and cost‐effectiveness of the facilitator‐enabled iSupport program. Phase 3 focused on embedding the iSupport program in health and aged care services.

## 2 Background

Dementia is the second leading cause of death and burden of disease in Australia. In a 2023 study, at least 140,000 family carers of PLWD were providing ongoing care in the community (Australian Institute of Health and Welfare 2024). Caring for PLWD can be physically and emotionally demanding. In Australia, 31% of carers of PLWD expressed feeling worried and depressed, with 6.7% diagnosed with a stress‐related illness (Australian Institute of Health and Welfare 2024). Studies continue to report unmet needs among carers of PLWD including dementia education, peer support, emotional support and access to respite services (Bressan et al. 2020; Steenfeldt et al. 2021). PLWD's conditions (i.e., changed behaviours and number of medications), level of dependency (i.e., physical function) and level of support carers received (i.e., PLWD's service levels) are also directly related to carers' sense of burden and stress (Connors et al. 2020). Carers often need to navigate the system to identify available services, financial support and carer rights. However, searching for and processing trustworthy, relevant information is time‐consuming and often stressful for carers (Bressan et al. 2020). Carers can also experience work‐care conflicts and social isolation due to care responsibilities, as well as changes in family dynamics when PLWD gradually lose their memory and communication ability, leading to carer grief (Steenfeldt et al. 2021). Carers of PLWD want ongoing support from health and social care professionals throughout the post‐diagnostic dementia care trajectory to reduce dementia care‐related stress (Yu, Hunter, et al. 2023; Yu et al. 2023b). However, carers are not typically viewed as clients of care service providers, and their support is often based primarily on the assessment of PLWD.

Recommendations for supporting carers of PLWD commonly include education, skill development, information provision, psychosocial support and physical support (Gao et al. 2022; World Health Organisation 2017). Building on these approaches, multicomponent psychoeducation interventions are considered effective in dementia care, as they aim not only to increase carers' knowledge but also to enhance coping skills, problem‐solving abilities and emotional regulation, thereby reducing behavioural and psychological symptoms in PLWD and alleviating carers' stress (Cheng et al. 2020; Trivedi et al. 2019). Theoretical mechanisms underpinning these interventions include fostering collaborative partnerships between carers and health professionals, supporting carers' active problem‐solving in response to care challenges, and encouraging self‐reflection to promote adaptive coping and resilience. By integrating these elements, such interventions are designed to empower carers, improve psychological well‐being, and facilitate sustainable caregiving practices.

The WHO iSupport for Dementia program has been implemented in diverse ways across global contexts. For example, previous studies in India and Portugal evaluated fully self‐guided versions of the program (Baruah et al. 2021; Teles et al. 2022). In contrast, a systematic review conducted by Yu et al. (2023b) found that staff‐facilitated psychoeducational interventions were associated with significant reductions in carers' depressive symptoms. Informed by these findings and building on the co‐design outcomes from Phase 1 of the project, the present study implemented the Australian version of iSupport for Dementia with facilitator support, in which trained facilitators guided carers through the program to enhance engagement and support. The iSupport facilitators in the present study were registered nurses from the two acute hospitals and registered nurse‐supported health and social care professionals employed by community aged care service providers. The role of the facilitator is not only to assist carers in learning about, navigating, accessing and utilising care services for PLWD, as indicated in the literature (Goeman et al. 2016), but also to build rapport with carers to offer them ongoing individualised support, including emotional support.

Virtual interventions and support have gained popularity over the past decade due to their flexible delivery, which eliminates the need for physical space. However, recent systematic reviews have indicated a lack of virtual psychoeducation and carer support, as well as inconsistent evidence regarding their outcomes and benefits (Yu et al. 2023a, 2023b). These limitations highlight the need for further research examining the effectiveness of virtual support models in dementia care.

Beyond intervention outcomes, there is limited evidence regarding the feasibility and fidelity of facilitator‐enabled dementia support programs. Previous systematic reviews reported that virtual interventions had a higher participant attrition rate than face‐to‐face interventions, with uncertain causes (Gonzalez‐Fraile et al. 2021). This highlights the importance of feasibility and fidelity studies to determine whether virtual interventions can be successfully implemented and sustained in real‐world settings.

Given their established roles in care coordination, education, psychosocial support and service navigation, nurses are well positioned to deliver and evaluate post‐diagnostic support interventions for PLWD and their family carers. Although nurse‐coordinated interventions have demonstrated positive outcomes across palliative care (Qtait et al. 2026), cardiac care (Miah et al. 2025) and cancer care (Molassiotis et al. 2021), evidence regarding nurse‐coordinated post‐diagnostic dementia support is particularly scarce in Australia. Furthermore, most international nurse‐led dementia care models have focused on post‐hospital discharge support, later‐stage dementia care, or the management of complex care needs, with limited attention to virtual psychoeducational support in the community. Consequently, a nursing‐specific knowledge gap exists regarding the feasibility, fidelity and potential value of nurse‐coordinated virtual interventions delivered during the transition from diagnosis to ongoing community‐based care. Therefore, this internal pilot mixed‐methods study evaluated the feasibility and fidelity of a facilitator‐enabled iSupport for Dementia program, generating evidence to inform nursing practice and the implementation of person‐centred post‐diagnostic dementia support services.

### 2.1 Study Aim

This internal pilot study aimed to assess the feasibility, fidelity and preliminary impact of a facilitator‐enabled iSupport for Dementia program for family carers of PLWD in the Australian context (Registration No. ACTRN12622000199718).

## 3 Methods

### 3.1 Study Design

**FIGURE 1:**
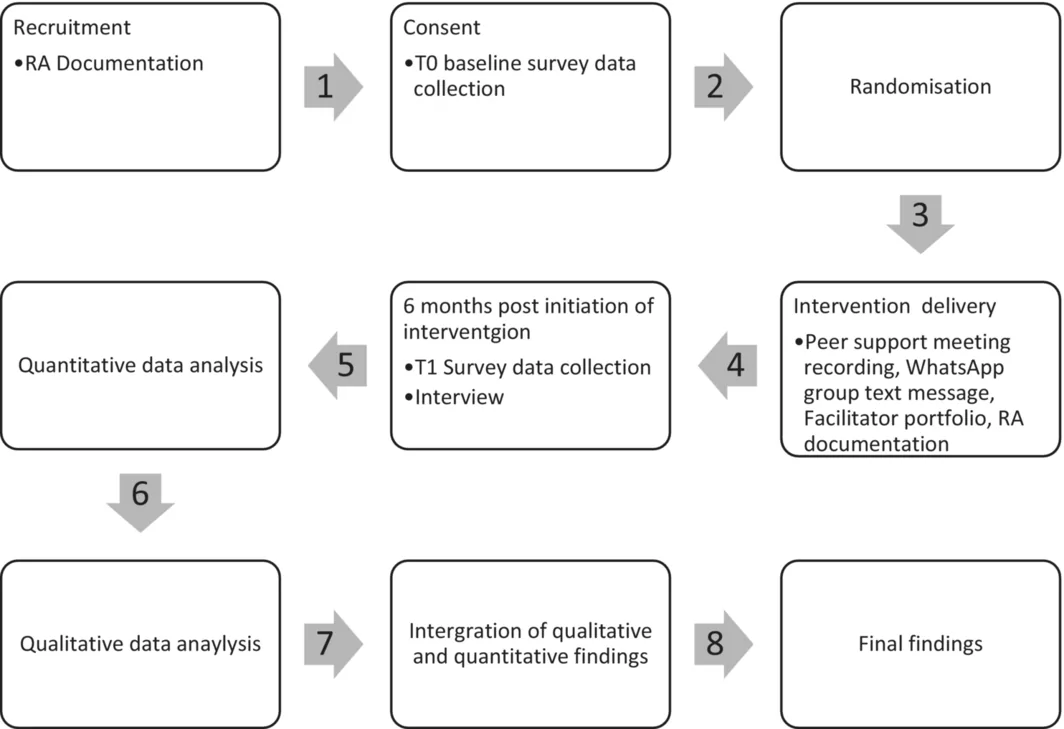
Study design and data collection point.

This study was a multi‐centred, convergent, mixed‐methods internal pilot study employing a parallel‐group, block RCT and a qualitative descriptive study design (see Figure 1). An internal pilot study is a pilot study embedded within the main study, with data used in both to address the recruitment and retention challenges of the RCT (Bond et al. 2023; Eldridge, Lancaster, et al. 2016). A convergent mixed‐methods design was chosen to facilitate the integrated interpretation of outcome data by combining quantitative and qualitative findings collected at the same time point. This approach enabled a more comprehensive understanding of the research problem, particularly where a single research method may be insufficient to address all aspects of the research questions. The internal pilot RCT design also enabled refinement of study procedures and intervention delivery to better align with participants' needs and real‐world clinical practice, thereby enhancing the feasibility and success of the main trial. We adhered to the Consolidated Standards of Reporting Trials 2010 statement and its extension for randomised pilot and feasibility trials (Eldridge, Chan, et al. 2016). We also reported according to Good Reporting of A Mixed Methods Study (GRAMMS) (O'Cathain et al. 2008) (Files S4 and S5).

### 3.2 Setting, Participants and Ethics Considerations

This study was conducted in two community aged care settings and two tertiary hospitals across Australia. Participants were enrolled between June 2022 and April 2023. Ethics approval was obtained from the Southern Adelaide Clinical Human Research Ethics Committee (no. 2021/HRE00273) and the Bolton Clarke Human Research Ethics Committee (no. 21007). Potential participants were assured that participating in the trial was voluntary and that they could refuse or withdraw without affecting the services they received.

#### 3.2.1 Inclusion and Exclusion Criteria

Family carers who meet the following criteria were invited to the study: (1) caring for a PLWD at home, but not necessarily co‐resident; (2) over 18 years of age and (3) for care recipients with no formal dementia diagnosis, but who present with observable signs and symptoms of (a) cognitive impairment; (b) declined self‐care ability and (c) behaviour change. Family carers who were involved in similar studies were excluded from the recruitment.

#### 3.2.2 Recruitment Procedure

The recruitment flyer was distributed to partner organisations, and a social media campaign, managed by the university media team, commenced in June 2022 to enhance recruitment. At the same time, at sites one, two and three, the research assistant supported two iSupport facilitators in screening organisations' historical data to identify potential participants. The screening at site four was conducted by clinical professionals rather than the research team due to local policy. Potential participants were contacted by a site‐employed research assistant via text message, email, or phone call to express their interest in participating. The research assistant then contacted potential participants who expressed interest in completing eligibility assessments and the PLWD's cognitive impairment stage assessment before consent. All participants received a paper‐based or electronic information pack, according to their preference, and had opportunities to discuss the study with the research assistant. The research assistant documented the recruitment process for data analysis. All participants in the RCT study received a $50 gift card each month for the duration of the study.

### 3.3 Interventions

**FIGURE 2:**
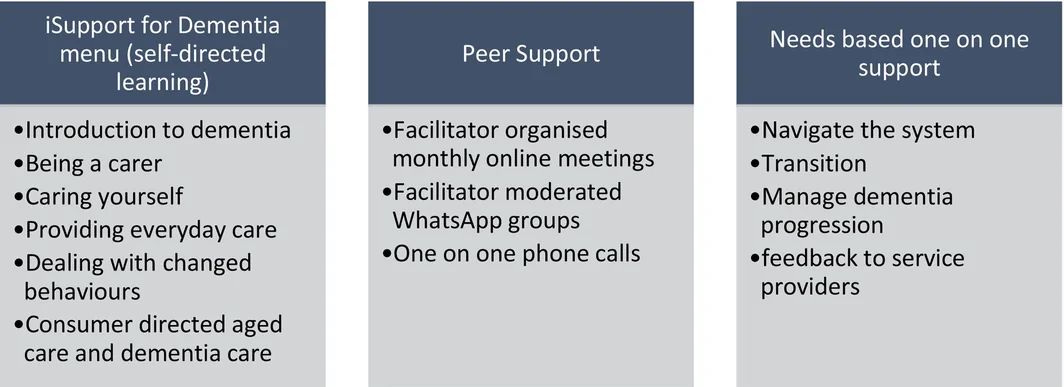
Intervention.

Carers were randomised into the intervention or usual care groups. Participants in the usual care group received publicly available carer resources, such as those available through the Dementia Australia website, without additional formal support from health professionals. Carers in the intervention group were supported by the iSupport facilitator and provided with the Australian iSupport for Dementia manual in either hard copy, electronic, or web‐based format, based on their preference. The Australian iSupport for Dementia manual contains six modules and 30 units. Carers were encouraged to choose 20 of the 30 units (70%) most relevant to them from the iSupport for Dementia manual to learn at their own pace. Carers were encouraged to contact the facilitator for support with managing transition, dementia progression‐related questions, or feedback to service providers. iSupport facilitators also created one or two private WhatsApp groups and peer support meetings for carers to exchange information. The planned peer support group size was 12 carers per group per site. Monthly online peer support meetings lasting 30 min were facilitated by iSupport facilitators to enable carers to interact with one another. The meeting was video‐recorded for carers in the same group to access. Carers participated in the main RCT for 12 months, whereas the internal pilot study data were analysed at 6 months for a proportion of participants. The interventions are displayed in Figure 2.

Each of the four industry partner sites employed a facilitator as an interventionist. The essential criteria for an iSupport facilitator are (1) currently employed as a health or social care professional and (2) experience in dementia care or aged care. iSupport facilitators were recruited from internal job advertisements within each participating organisation. Four facilitators were employed at the beginning of the project: two from hospitals were registered nurses, and two from community aged care services were social care professionals.

### 3.4 Intervention Fidelity

All facilitators were required to familiarise themselves with the Australian iSupport for Dementia manual, undertake 2 days of online training with the research team, and receive an iSupport implementation manual. The implementation manual contains dementia care‐related case scenarios and research procedures for facilitators to do self‐directed learning before the commencement of recruitment. The facilitators were also required to attend biweekly online facilitator meetings to share their experiences, as described in the study protocol including the difficulties they encountered while delivering the required intervention and the strategies they used to address them. The project leader chaired the regular meetings as part of strategies to monitor the intervention's fidelity. iSupport facilitators were requested to submit a monthly portfolio reflecting their project activities and experiences.

### 3.5 Sample Size

For the pilot study, there is no consensus on the minimum sample size, although recommendations commonly suggest between 12 and 30 participants per group (Eldridge, Chan, et al. 2016). The present internal pilot study targeted 30 carers per arm, with a total sample size of 60 participants considered sufficient to assess feasibility, recruitment, retention, intervention fidelity, and to provide preliminary estimates for planning the definitive trial.

### 3.6 Randomisation

Each participant was assigned a unique code in the study. A biostatistician conducted the randomisation and had not been involved in recruitment nor knew the participants. The personal code, the participant's relationship with the PLWD and the PLWD's stage of cognitive impairment were sent to the biostatistician. The randomisation result was then communicated to the facilitator and the research assistant for follow‐up. A randomly generated block size of four was used to allocate carers to one of the two groups at each recruitment site, ensuring the two groups were of equivalent size. The randomisation ensured an equivalent distribution of spouse versus non‐spouse carers and PLWD with mild versus moderate cognitive impairment in each intervention group. Due to the nature of the intervention, it is impossible to conceal it from the participants, the research assistant and the iSupport facilitators.

### 3.7 Outcome Evaluation and Instruments

#### 3.7.1 Estimated Recruitment, Retention and Attrition Rate

The feasibility of the study was evaluated by estimating the recruitment, retention and attrition rates. The recruitment rate in this pilot study was defined as the proportion of participants who were enrolled in the RCT among those approached by the research team and eligible to participate. The retention rate was the percentage of participants who remained in the program at the 6‐month mark, whereas the attrition rate was the percentage of participants who left the program at the same time.

#### 3.7.2 Study Fidelity

The study's fidelity was measured by carers' engagement in the monthly peer support group meetings, as well as their support requests and delivery, and their completion rate of the iSupport manual in the intervention group. The facilitator's support for carers, as per protocol, was also measured using the carers' satisfaction survey. The survey included 10 questions covering the support provided by the iSupport facilitators and was rated on a 5‐point Likert scale (see File S1). Protocol adherence was evaluated by analysing the documented facilitators' portfolios.

#### 3.7.3 Preliminary Impact at 6 Months

The outcome measures designed for the main trial were also used for the pilot trial. This pilot study measured the carer's quality of life (QOL), the PLWD's QOL, the carer's self‐efficacy, the carer's perceived quality of support, the PLWD's changed behaviour frequency, and the carer's response to the behaviour. The instrument details, scoring, validity and reliability are displayed in File S2.

### 3.8 Data Collection

Recruitment data were collected on the number of people screened via historical datasets, the number of people who met the selection criteria, the number of people assessed for eligibility, and the proportion of eligible people who agreed to participate. The research assistant documented participants who withdrew from the study earlier. The research team also predetermined that data collection would cease if the PLWD was admitted to a long‐term aged care facility or passed away. Carers who withdrew from the study were encouraged to disclose their reasons for withdrawal, but it was not mandatory to do so to respect their privacy.

Participants' demographic data were collected at the baseline. Data used to evaluate the effectiveness of the intervention were collected through baseline and 6‐month post‐intervention surveys. Participants were offered different formats for survey data collection, including paper‐based, online, or phone‐based, with the research assistant selecting the format based on participants' preferences. The paper‐based survey was posted to the carer's nominated address with a pre‐paid, pre‐addressed envelope and the research assistant's phone number. The online survey was built in Qualtrics (Qualtrics 2005) via the university server for data security. Survey data collection ceased if the PLWD was admitted to permanent residential care or passed away. Qualitative data were downloaded, transcribed, and reported descriptively including survey comments, virtual peer support meeting recordings, WhatsApp group text messages, the facilitator's portfolio and research assistant records. Carers in the intervention group, site leaders, and facilitators were invited to an interview 6 months after the intervention had been implemented. The semi‐structured interview was voluntary, and consent was obtained; it lasted 40–60 min. The interview was audio‐recorded for data analysis. The semi‐structured interview guide is provided in File S3.

### 3.9 Data Analysis

#### 3.9.1 Quantitative Data Analysis

Descriptive statistics were used to summarise feasibility outcomes and demographic characteristics, with means and standard deviations (SD) for continuous variables and frequencies and percentages for categorical variables. The recruitment rate was calculated as the proportion of consented and randomised carers among those who were eligible and approached. Consistent with the exploratory nature of a pilot study, analyses of intervention outcomes were primarily descriptive and interpreted with caution. An intention‐to‐treat approach was used, maintaining participants in their allocated groups. Prior to conducting parametric tests, the distributions of continuous variables were assessed visually using histograms and Q‐Q plots. Between‐group comparisons at baseline and 6 months were conducted using independent‐samples *t*‐tests for normally distributed continuous variables, Wilcoxon rank‐sum tests for non‐normally distributed variables and chi‐squared tests for categorical variables. Missing outcome data were handled using multiple imputation by chained equations (MICE). A total of 10 imputed datasets were generated, incorporating all variables included in the analysis models, as well as relevant baseline covariates to improve the plausibility of the imputations. The imputation procedure assumed that data were missing at random (MAR). Estimates from the imputed datasets were pooled according to Rubin's rules. Given the exploratory nature of this pilot study, the imputation approach was intended to provide preliminary estimates rather than definitive inference. All tests were two‐sided, with 95% confidence intervals (CI) reported. *p*‐values are presented for exploratory purposes only, and no adjustment for multiple comparisons was applied; therefore, findings should be interpreted with caution due to the increased risk of Type I error. Analyses were conducted using IBM SPSS Statistics 28 (IBM Corp. 2021).

#### 3.9.2 Qualitative Data Analysis

Audio‐recorded data were transcribed verbatim using the online Microsoft Word transcription function. The first author also listened to the audio recordings and corrected the transcriptions for analysis, and the second author checked the transcriptions. A deductive qualitative analysis suggested by Love and Corr (2021) was applied. A coding framework was developed based on the study outcome measure. The author coded data using pre‐developed codes based on the outcome measure; then, similar codes were grouped into themes. The codes and themes were reviewed by team members and then refined to address their feedback.

#### 3.9.3 Mixed Methods Data Analysis, Interpretation and Integration

After the first step of quantitative and qualitative data analysis, a joint display table was created to compare the two datasets and examine their similarities and differences. During the comparison, similarities and differences were identified. Quantitative findings were grouped under the study's aims and objectives (i.e., feasibility, fidelity and effectiveness of the program). The quantitative findings were transformed into qualitative descriptive findings based on Sandelowski (2000) data transformation strategies for further integration. The integrated and interpreted quantitative and qualitative data were presented as findings in the results section of this report and then further synthesised in the discussion section.

## 4 Results

### 4.1 Characteristics of the Participants

**FIGURE 3:**
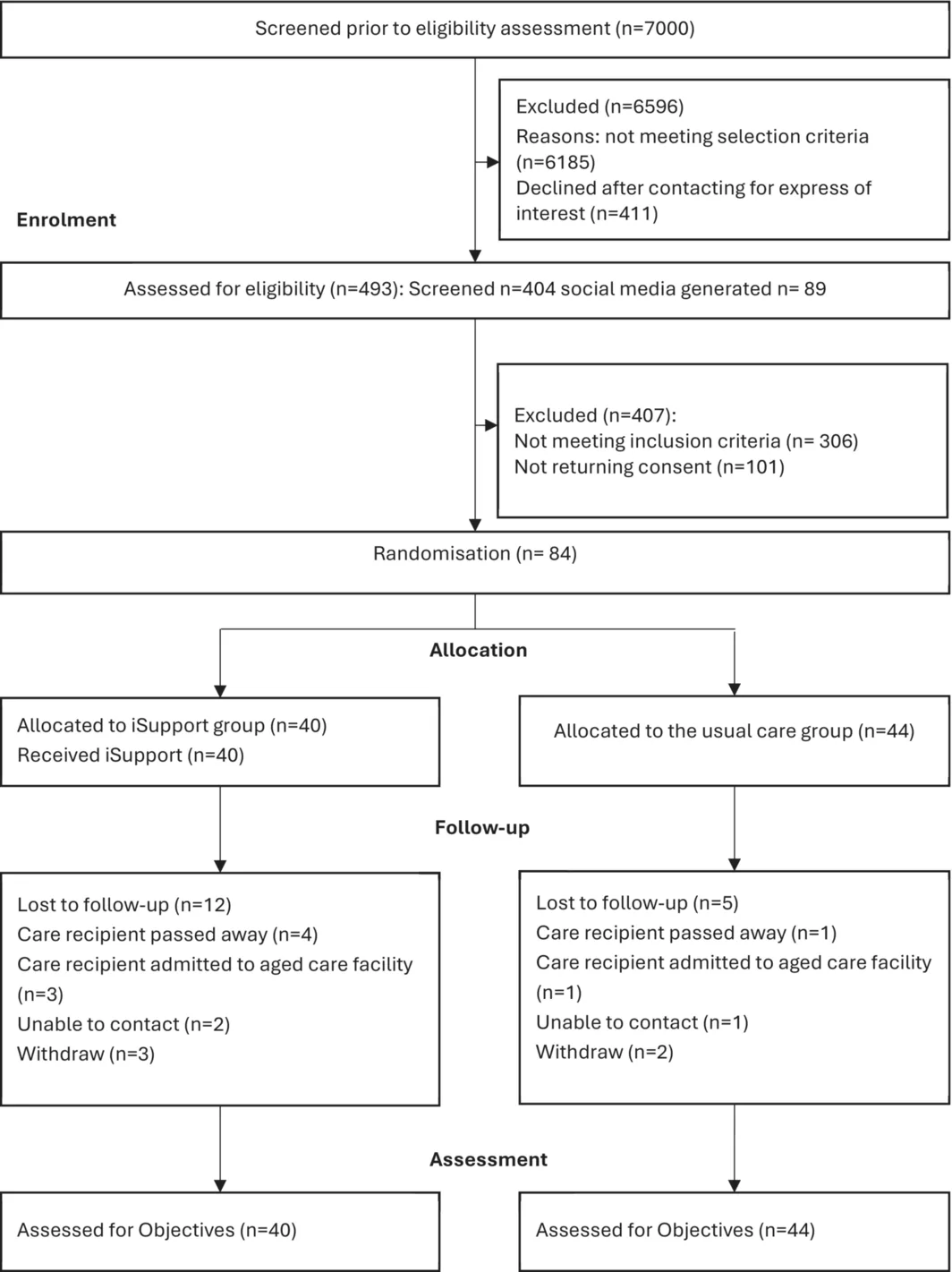
Flowchart of recruitment and retention.

Eighty‐four carers were enrolled in this internal pilot study (see Figure 3). Of those, 40 were allocated to the intervention group and 44 to the usual care group. Table 1 presents the baseline characteristics of participants. The average age of the family carers was 66 years (SD = 14). The majority were female (*n* = 57, 68%), spouses (*n* = 45, 54%), retired (*n* = 49, 60%) and lived in the same household as the PLWD (*n* = 59, 70%). More carers in the intervention group lived in the same household than the usual care group. Family carers spent 10 h (SD = 11) per day and nearly 6 days (SD = 2) per week on care activities. Carers in the intervention group spent more days on care activities than those in the usual care group. Approximately 82% (*n* = 67) of carers have some family support.

**TABLE 1:** Family carers' socio‐demographic characteristics at baseline.

Demographics	Intervention group (*N* = 40)	Usual care (*N* = 44)
Age, mean (SD)	66 (14)	66 (13)
Gender, *n* (%)		
Male	14 (35)	13 (30)
Female	26 (65)	31 (71)
Relationship, *n* (%)		
Spouse	20 (50)	22 (50)
Non‐spouse	20 (50)	22 (50)
Marital status, *n* (%)		
Married/partner/de‐facto	32 (80)	37 (84)
Single/divorced/widow	7 (18)	7 (16)
Employment status, *n* (%)		
Employed	15 (38)	14 (33)
Unemployed	2 (5)	2 (5)
Retired	23 (58)	26 (62)
Live in the same household as PLWD, *n* (%)		
Yes	32 (80)	27 (61)
No	8 (20)	17 (39)
Years living in the same household as PLWD, mean (SD)	38 (23)	42 (21)
Average hours per day on carer activities, mean (SD)	10 (10)	9 (11)
Average days per week on carer activities, mean (SD)	6 (1)	5 (2)
Years of being in a carer's role mean (SD)	4 (2)	5 (5)
Do other family members support you in your caring role, *n* (%)		
Yes	33 (83)	34 (81)
No	7 (18)	8 (19)

Abbreviation: PLWD, a person living with dementia.

The average age of PLWD was 80 years (SD = 11). The number of males (*n* = 41, 49%) and females (*n* = 42, 51%) was similar. The majority of the PLWD presented with mild cognitive impairment (*n* = 55, 66%) and had some but fewer than five types of chronic conditions (*n* = 49, 58%). The average duration of dementia diagnosis or observation of cognitive impairment symptoms was 4 years (see Table 2).

**TABLE 2:** Person living with dementia's socio‐demographic characteristics.

Demographics	Intervention group (*N* = 40)	Usual care group (*n* = 44)
Age, mean (SD)	80 (8)	80 (13)
Gender, *n* (%)		
Male	20 (50)	21 (49)
Female	20 (50)	22 (51)
Cognitive stage, *n* (%)		
Mild	37 (68)	28 (64)
Moderate	13 (33)	16 (36)
Chronic condition, *n* (%)		
No	5 (14)	7 (21)
1–4	27 (77)	22 (67)
≥ 5	3 (8)	4 (12)
Length of diagnosis or show symptoms, mean (SD)	5 (3)	4 (4)

Nineteen individual interviews were conducted with 13 carers, three facilitators, and three site leaders. Table 3 displays the characteristics of the participants who attended the interviews.

**TABLE 3:** Characteristics of participants in the interview.

	Staff	Carers
	Site leaders *n* = 3	Spouse *n* = 7
	Facilitators *n* = 3	Children *n* = 6
Age		
20–40	1	1
41–65	4	5
> 65	1	7
Gender		
Male	1	2
Female	5	11
Education level	Diploma *n* = 1 Bachelor *n* = 1 Masters *n* = 1 PhD *n* = 3	N/A
Units completed in the iSupport manual	N/A	All *n* = 5 80% *n* = 3 70% *n* = 2 < 50% *n* = 3
Role in the organisation	Project officer *n* = 2 iSupport lead *n* = 1 Director *n* = 1 Unit manager *n* = 1 Senior research fellow *n* = 1	N/A
Working status	N/A	Full‐time worker *n* = 2 Part‐time worker *n* = 1 Casual worker *n* = 1 Retired with casual work *n* = 1 Retired *n* = 7 Unemployed due to caring role *n* = 1
Length in the current role		
1–11 months	2	N/A
1–4 years	1	
5–9 years	3	
> 10 years	1	
Length in the carer role		
1–4 years	N/A	9
5–9 years		3
> 10 years		1
Length of employment in the organisation		
1–11 months	1	N/A
1–4 years	2	
5–9 years	2	

We also downloaded and analysed 24 recorded peer support meetings, 32 facilitators' portfolios, 97 survey comments, WhatsApp messages between October 2022 and October 2023, and research assistant documentation from March 2022 to April 2023. With permission, a letter from one carer summarising their experiences was also included in this analysis. Selected quotes were given a code based on the data source. For example, the recording of a peer support meeting was coded as G_P and the WhatsApp messages were coded as G_T. Survey comments were coded as SC, research assistants' documents were coded as RA, and the facilitator portfolio was coded as FP.

### 4.2 Integrated Finding 1: Feasibility, Retention and Attrition of the Study

Eighty‐four carers consented to participate and were randomly assigned to either the intervention group or the usual care group. The recruitment rate was 10%, as detailed below. We screened four historical databases and identified approximately 7000 potential carer participants, and an additional 89 carers expressed interest via social media. Of these, 815 carers met the inclusion criteria and were approached for participation; this group constituted the denominator for the recruitment rate. Among those eligible and contacted, over 50% (*n* = 411) declined to participate, 306 refused following the initial assessment, and a further 101 carers who initially agreed to participate did not return consent forms or withdrew prior to randomisation. This resulted in an overall recruitment rate of approximately 10% of eligible carers approached.

The screening process took longer than anticipated because care recipients often lacked a clearly identifiable dementia diagnosis, necessitating a detailed review of case notes [RA]. Eligibility assessment and consent procedures were also time‐consuming, frequently involving multiple phone calls, each lasting 10–60 min [RA]. Returning consent forms could take several weeks to 3 months, with some carers requiring multiple reminders [RA, FP3_2]. Contacting participants was further complicated by reluctance to answer calls from unknown numbers, particularly among older carers [I_F3].

The reasons for carers declining the study include uncertainty of which group they may be allocated to (i.e., a preference for intervention or usual care group) [RA]; the iSupport program does not meet their needs (e.g., the carer needed a financial advisor at the time) [RA]; too busy to participate [RA]; wanted research team to contact their children (Children were not contactable) [RA], already well supported [FP3_2], not interested in Zoom meeting [FP3_2], overwhelmed by the paperwork for consent and monthly surveys [RA], and has difficulty in reading or accessing the internet [FP3_2].

Of the 84 participants, 17 were lost to follow‐up at the 6‐month follow‐up, resulting in an overall attrition rate of 20% (17/84). Retention at 6 months was 70% in the intervention group (28/40) and 89% in the usual care group (39/44), corresponding to attrition rates of 30% and 11%, respectively. Attrition was higher in the intervention group (30%, 12/40) than in the usual care group (11%, 5/44). Of the 12 participants who dropped out of the intervention group, more were from community aged care services (*n* = 8) than from hospitals (*n* = 4). The reasons for the 17 participants dropping out included the passing away of PLWD (*n* = 5), PLWD being admitted to a permanent residential care facility (*n* = 4), being unable to contact (*n* = 3) and withdrawing from the study without disclosing a reason (*n* = 5).

### 4.3 Integrated Finding 2: Intervention Fidelity

All carers in the intervention group (*n* = 40) received either a hard copy of the iSupport manual, an electronic book, or access to the website once it became available. At the 6‐month follow‐up, 25 of 28 (89%) carers in the intervention group documented their manual completion. Of those who reported completion, 23 (92%) engaged with some parts of the book. Only two (2/25, 8%) carers did not read any part of the book. Qualitative data analysis indicated that most carers engaged with the iSupport hardcopy manual with facilitators' support. For example, one carer stated in the peer support meeting: ‘I read module four because you [facilitator] asked us to, which was helpful. It confirmed many things I had picked up along the way [G4_P]’. This finding indicated that although some carers may not have completed the required proportion of the manual, the information in the manual was delivered by facilitators in various formats and may have affected their understanding of dementia.

Among carers who completed follow‐up (*n* = 28), 22 (79%) requested support from their facilitators, whereas six (21%) did not. Among those who received support, 15 (68%) reported being satisfied (File S2). Facilitators offered support in different ways. For example, one facilitator encouraged her group to document their loved one's favourite things on ‘Capturing Memories’ [FP3_2]. The carer also requested one‐on‐one support from the facilitator for various reasons. All facilitators received requests for one‐on‐one support from carers at least twice a month, and some received up to 8 phone calls or emails per month. ‘The time spent by the facilitator for each support varied from 20 to 60 min’ [FP1_2, FP2_2, FP3_2, FP4].

Online peer support meetings were designed to run for 30 min each month and were organised by iSupport facilitators. Each meeting was attended by 4–8 carers. However, when attendance was higher, facilitators reported that sessions often exceeded the planned duration: ‘some meetings ran over 30 min, and most ran for 1 h due to many carers having difficulty logging in at the beginning or wanting to chat more after 30 min’ [FP1_2, FP2_2]. Compared to the planned peer support meeting length (12 carers/30 min/session = 2.5 min/carer), the delivered meetings allowed substantially more time per participant (e.g., 8 carers per 60‐min session or 4 carers per 30‐min session, equivalent to approximately 7.5 min per carer). According to the facilitator portfolio, most participants attended 80% of the peer support meetings. Not everyone in the study was willing to participate in the monthly Zoom peer support meetings. For example, one site has four participants who opt out of Zoom meetings but are happy for the facilitator to contact them via phone monthly [FP1_2].

iSupport facilitators created five private WhatsApp groups for carers in the intervention group. Site one created two WhatsApp groups to limit the number in each group. Most groups have caregivers who regularly interact, whereas others remain in the group but do not engage. Carers used WhatsApp messages to exchange information and offer support to one another. The information exchanged includes ‘how to access services’ [G2_T], ‘nutrition’ [G2_T], ‘how to respond to changed behaviours’ [G1_T] and ‘ensure other carers were doing ok’ [G3_T]. On one occasion, the facilitator was on leave, and carers in the group also texted to ‘make sure the facilitator was okay’ [G4_T]. Facilitators and research assistants observed an increase in interactions within the WhatsApp group over time. For example: ‘More interaction observed between carers in WhatsApp groups’ [RA]. These findings indicate that the use of WhatsApp among carers of PLWD varies and that it may take longer for carers to become comfortable interacting with one another. Intervention fidelity is displayed in Table 4.

**TABLE 4:** Intervention fidelity.

Outcome	Description	*n*/*N* or format	%/Value
Intervention group total		40	—
Retention	Completed 6‐month follow‐up	28/40	70%
iSupport for dementia manual	Completed manual (among those followed up)	25/28	89%
	Read some parts (among those completing the manual)	23/25	92%
	Non‐engagement	2/25	8%
Facilitator support	Requested support	22/28	79%
	Did not request support	6/28	21%
	Satisfied with support (among those who received it)	15/22	68%
Peer support meetings (planned)	Session duration	30 min	—
	Number of cares per session	12	—
	Time per care	2.5 min/carer	—
Peer support meetings (actual)	Session duration	30–60 min	—
	Number of cares per session	4–8	—
	Time per care	7.5 min/carer	—
WhatsApp groups	Number of groups	5	—
	Group organisation	One site created 2 groups to reduce the size	—
	Engagement	Mixed	—

### 4.4 Integrated Finding 3: Preliminary Intervention Impact

There were no clear differences in any measure between the intervention and usual care groups at baseline (Table 5). After 6 months of intervention, effect size estimates suggested a large reduction in behavioural problems (RMBPC‐F) in the intervention group compared to usual care (*d* = 0.93), alongside a moderate improvement in caregiving self‐efficacy for responding to behavioural symptoms (*d* = 0.59). Most other outcomes demonstrated small or negligible effects. In contrast, a large effect was observed for self‐efficacy in controlling upsetting thoughts (*d* = 1.05), favouring the usual care group (Table 6). Qualitative findings provide context for these quantitative trends. Carers and facilitators in the intervention group described active engagement in identifying, anticipating and managing behavioural changes in PLWD. The iSupport program appeared to promote proactive problem‐solving and reflective caregiving practices. For example, facilitators provided timely, situation‐specific guidance, such as maintaining routines during disruptions, which carers found helpful in managing potential behavioural changes. For example, one carer had tradespeople working around the house, with the iSupport facilitator posting a message on WhatsApp to offer some tips to alleviate a potential increase in the frequency of changed behaviours during this time: ‘It may help to remember that you are doing the right thing by keeping as much to the routine as possible while these disruptions happen…’ [G4_T].

**TABLE 5:** Outcome at baseline between groups.

Outcomes	Intervention (*n* = 40)	Usual care (*n* = 44)	MD (95% CI)	Cohen's *d*	95% CI (*d*)
	Mean (SD)	Mean (SD)			
SF12‐PCS	51.98 (8.96)	50.83 (10.74)	1.15 (−3.30 to 5.59)	0.12	−0.31 to 0.54
SF12‐MCS	43.57 (9.68)	44.46 (10.27)	−0.90 (−5.36 to 3.57)	−0.09	−0.52 to 0.34
QOL‐AD	2.20 (0.56)	2.23 (0.54)	−0.03 (−0.48 to 0.41)	−0.05	−0.48 to 0.37
RMBPC‐F	2.51 (0.97)	2.41 (0.89)	0.10 (−0.44 to 0.64)	0.11	−0.32 to 0.54
RMBPC‐R	1.99 (0.45)	2.05 (0.40)	0.05 (−0.20 to 0.30)	−0.14	−0.57 to 0.29
QOS	2.60 (0.16)	2.74 (0.19)	−0.14 (−0.40 to 0.12)	−0.79	−1.24 to −0.35
SE‐OR	46.69 (14.46)	47.1 (14.16)	−0.41 (−21.28 to 20.45)	−0.03	−0.46 to 0.40
SE‐RB	69.82 (4.42)	70.57 (5.65)	−0.75 (−8.15 to 6.65)	−0.15	−0.58 to 0.28
SE‐CT	57.65 (5.04)	60.06 (3.56)	−2.41 (−8.78 to 3.96)	−0.56	−0.99 to −0.12

Abbreviations: CI, confidence interval; CT, controlling thoughts; MCS, mental component score; MD, mean difference; OR, obtaining respite; PCS, physical component score; QOL‐AD, quality of life in Alzheimer's disease; QOS, quality of social support; RB, responding to behaviours; RMBPC, Revised Memory and Behaviour Problem Checklist; SE, self‐efficacy domains; SF12, 12‐item Short‐Form Health Survey.

**TABLE 6:** Outcomes at baseline and 6 months with within‐ and between‐group differences.

Outcome	Group	Baseline	6‐month	Within‐group	*p* *	Between‐group	*p* *	Cohen's *d*
		Mean (SD)	Mean (SD)	MD (95% CI)		MD (95% CI)		
Carer's QOL_SF12 PCS	IG	51.98 (8.96)	52.62 (10.66)	−0.50 (−3.55 to 4.55)	0.80	1.06 (−4.30 to 6.40)	0.69	0.10
	UG	50.83 (10.74)	51.56 (10.84)	−1.08 (−6.52 to 4.36)	0.69			
Carer's QOL_SF12 MCS	IG	43.57 (9.68)	45.88 (11.04)	−1.18 (−7.03 to 4.67)	0.68	2.53 (−3.20 to 8.20)	0.38	0.22
	UG	44.46 (10.27)	43.36 (11.88)	1.02 (−4.02 to 6.06)	0.68			
QOL_AD	IG	2.20 (0.56)	2.12 (0.59)	0.07 (−0.05 to 0.19)	0.21	−0.04 (−0.50 to 0.50)	0.87	0.07
	UG	2.23 (0.54)	2.16 (0.55)	0.04 (−0.03 to 0.11)	0.22			
RMBPC‐F	IG	2.51 (0.97)	1.77 (0.44)	0.01 (−0.07 to 0.09)	0.72	−0.76 (−1.20 to −0.30)	0.00	0.93
	UG	2.41 (0.89)	2.53 (0.99)	−0.12 (−0.21 to −0.02)	0.02			
RMBPC‐R	IG	1.99 (0.45)	1.77 (0.44)	0.22 (0.14 to 0.30)	< 0.001	−0.16 (−0.40 to 0.10)	0.00	0.00
	UG	1.99 (0.45)	1.77 (0.44)	0.22 (−0.05 to 0.45)	0.19			
Quality of social support	IG	2.60 (0.16)	2.63 (0.12)	−0.03 (−0.18 to 0.11)	0.55	−0.03 (−0.20 to 0.20)	0.75	0.22
	UG	2.74 (0.19)	2.66 (0.15)	0.07	0.21			
Caregiving self‐efficacy for obtaining respite	IG	46.69 (14.46)	52.77 (16.50)	−6.09 (−8.86 to 3.21)	< 0.001	3.74 (−21.10 to 28.50)	0.74	0.22
	UG	47.10 (14.16)	49.03 (17.51)	−1.93 (−8.08 to 4.21)	0.43			
Caregiving self‐efficacy for responding to changed behaviours	IG	69.82 (4.42)	76.01 (5.77)	−6.19 (−12.56 to 0.18)	0.05	2.84 (−3.90 to 9.60)	0.05	0.59
	UG	70.57 (5.65)	73.18 (3.08)	−2.60 (−6.33 to 1.12)	0.12			
Caregiving self‐efficacy for controlling upsetting thoughts	IG	57.65 (5.04)	57.93 (4.37)	−0.29 (−7.65 to 7.08)	0.92	−4.07 (−9.50 to 1.30)	0.12	1.05
	UG	60.06 (3.57)	62.03 (3.06)	−1.95 (−0.52 to 4.63)	0.46			

*Note:* No adjustment for multiple comparisons was applied; therefore, results should be interpreted with caution due to the potential for Type I error.

Abbreviations: CI, confidence interval; IG, intervention group; MD, mean difference; RMBPC‐F, Revised Memory and Behaviour Problem Checklist frequency; RMBPC‐R, Revised Memory and Behaviour Problem Checklist reaction; SF12‐MCS, QOL: 12‐item Short‐Form Health Survey mental health component score; SF12‐PCS, QOL: 12‐item Short‐Form Health Survey physical health component score; UG, usual care group.

* *p*‐values are reported for exploratory purposes only and are not interpreted as indicators of statistical significance.

No meaningful changes were observed in PLWD QOL, carers' QOL, or self‐efficacy related to emotional regulation, consistent with the small effect sizes in these domains. Nevertheless, qualitative data indicated that carers valued the emotional support provided by facilitators, which may not have been fully captured by the quantitative measures. For example, one carer said: ‘The most significant thing was emotional support [from facilitators]…she was caring … she gave me lots of positive thoughts, so emotional support and positive thoughts’ [I_C13]. Both groups reported above‐average levels of social support at baseline and 6 months, with no clear between‐group differences. However, descriptive trends suggested slight improvements in perceived support among intervention participants, whereas the usual care group showed a slight decline. Qualitative findings suggest that facilitator support helped carers navigate service systems and access resources, particularly in addressing practical challenges related to respite care and service entitlements. One carer stated:

The facilitator [aged care service provider] helped me get a code number for respite for my husband; the difficulty was getting a test number when you already have a package. She [the facilitator] sent me the literature, which showed that I could get just a number because I used up all my packages. That was a practical thing that [facilitator] helped me with. [I_C8]

## 5 Discussion

This internal pilot study aimed to assess the feasibility and explore the preliminary impact of a facilitator‐enabled flexible iSupport for Dementia program for informal carers. The strength of this pilot study lies in its use of a mixed‐methods design, which allowed quantitative and qualitative findings to validate and confirm one another, thereby enhancing the understanding of the results. Moreover, an internal pilot RCT design allowed justification of the intervention, ensured the main RCT was more acceptable and suitable for the clinical area, and reduced recruitment pressure.

Recruiting family carers of PLWD in the community proved challenging, consistent with previous findings (Heward et al. 2022; Walters et al. 2017). Lessons from our pilot study highlight the importance of carefully assessing the target population's social and logistical circumstances to enhance program uptake. The recruitment process itself may place an additional burden on carers, potentially discouraging participation. Initially, the study aimed to recruit family carers of individuals with a formal diagnosis of mild or moderate dementia, with eligibility determined through assessments of PLWD conducted by a research assistant. Additionally, carers were required to be computer users with internet access. These criteria posed significant barriers, particularly for older carers who may have limited technological proficiency and greater social isolation, thereby requiring additional support. Conversely, younger carers, who are more likely to be employed, well supported, and technologically literate, may perceive the program as not fully meeting their needs. In the first 2 months of unsuccessful recruitment, after receiving feedback from the research assistant and site leaders, the inclusion criteria were expanded to include carers of people with any level of cognitive impairment. The assessment of PLWD's level of impairment was modified to incorporate carers' observations, thereby reducing the burden on carers, as many PLWD require substantial encouragement and effort from carers to participate in assessments conducted by research assistants.

Our study also observed a higher dropout rate in the intervention group and among participants recruited from community aged care services. Most dropouts were due to care recipients passing away or transitioning to residential aged care. This pattern likely reflects the more advanced stage of the dementia trajectory among community‐recruited participants compared to those recruited from acute hospital settings, especially given that waiting times for community services in Australia can extend up to a year. In addition to disease progression, perceived burden appears to have contributed to dropout. Interview findings indicated that some carers found the intervention overly demanding given their caregiving responsibilities, limited time, and emotional strain. Practical barriers, including challenges related to technology use and scheduling, further limited engagement. Modifications to improve accessibility, such as removing the requirement for computer use and offering telephone‐based support alongside hard‐copy materials, partially addressed these issues but also altered the intervention's nature, making it a flexible format. Taken together, these findings underscore the importance of aligning intervention intensity and delivery with carers' capacity, and of carefully considering recruitment pathways and timing in relation to the dementia trajectory in future trials.

Characteristics of the RCT design can also pose barriers to participant enrolment and are not easy to address (Naidoo et al. 2020; Rodríguez‐Torres et al. 2021). Our study, along with others, supports the notion that the complexity of the information provided to participants and follow‐ups was burdensome for some potential participants and may have influenced their decisions (Baker et al. 2021; Naidoo et al. 2020). Although certain core components of RCTs are not easily adaptable, broadening eligibility criteria and increasing the flexibility of the intervention have been widely discussed in the literature as ways to reduce barriers to participation (Briel et al. 2016; Rodríguez‐Torres et al. 2021). In our study, recruitment improved when participants were offered greater flexibility and autonomy. This includes modifying inclusion criteria, providing iSupport for Dementia in hardcopy books, and allowing participants to opt out of online meetings in favour of telephone‐based support from a facilitator. Nevertheless, it is important to recognise that not all individuals who meet eligibility criteria will perceive such programs as relevant or feasible, reflecting broader social, logistical and perceptual factors that shape engagement. Some carers may face constraints such as limited time, competing responsibilities, or technological barriers, whereas others may feel they already have sufficient support and therefore perceive little need for additional interventions. These considerations highlight that the effective target population for iSupport may be narrower than the eligible population, and underscore the need for complementary strategies to reach and support carers who are unable or unwilling to engage with this type of program.

We found that the facilitator‐enabled iSupport for Dementia program was feasible. Participants reported good engagement with the program, reflected in relatively high attendance. Compared with other RCTs involving people with chronic conditions, which report retention rates of 82%–100% (Harris et al. 2021; Sui et al. 2023), retention in this study was lower but comparable to or slightly higher than rates reported in similar trials involving carers of people living with dementia (Baruah et al. 2021; Cristancho‐Lacroix et al. 2015). The lower attrition rate in this study may be attributed to the internal pilot study design, which allows for the modification of interventions to better fit real‐world practice (Loudon et al. 2015).

Among all intervention components, facilitator support was favoured by participants. Our findings support a previous study showing that carers of PLWD prefer health professional‐led peer support (Yu, Hunter, et al. 2023). In the present study, carers appreciated both online and telephone‐based support from the facilitator, consistent with evidence suggesting that telehealth and online modalities can positively impact carers' wellbeing (Saragih et al. 2022). This model differs from traditional roles of key workers or support workers, which typically rely on face‐to‐face interactions (Goeman et al. 2016; Reilly et al. 2015). Although remote support can reduce logistical burdens such as travel and the need for physical space, some carers still prefer in‐person contact. This suggests that a blended approach, combining face‐to‐face and remote support, may better accommodate the diverse needs and preferences of carers. In addition, most participants welcomed online peer support meetings as opportunities to exchange information and provide mutual support. This is consistent with previous findings highlighting the importance of experiential knowledge sharing in meeting carers' needs and enhancing their understanding (Carter et al. 2020). However, ongoing technical support was required to ensure carers were confident and competent in using online platforms. This finding is consistent with a previous study by Banbury et al. (2019). Our study also found that the duration of peer support meetings influenced carers' experiences, indicating that flexibility in meeting length and scheduling may further enhance engagement and enable more effective interaction among participants.

We found that some but not all carers in this study used WhatsApp groups to exchange information. Previous studies have shown that online chat groups, such as WhatsApp, have become the leading platform for online dementia education in the Chinese American community (Shu and Woo 2020). Carers also use online platforms or social media to form supportive communities (Johnson et al. 2022). Our findings indicated that carers benefited from facilitator‐moderated online chat groups via WhatsApp, but were also cautious about the interaction. Some carers in our study did not use WhatsApp because they believed that relationships must be built before exchanging information on social media. This finding is similar to the study by Johnson et al. (2022), which found that carers and PLWD were motivated to use social media to connect with others but were also challenged by privacy and publicity concerns. This finding suggests that carers may require more time to establish a trusting relationship with this type of peer support, allowing them to share their life experiences.

In this pilot study, the facilitator‐enabled iSupport program demonstrated potentially favourable trends in selected outcomes. At 6 months, effect size estimates indicated a large reduction in behavioural problems (RMBPC‐F; *d* = 0.93) and a moderate improvement in carers' self‐efficacy in responding to behavioural symptoms (*d* = 0.59). In contrast, most other outcomes, including QOL and social support, demonstrated small or negligible effects. A large effect was also observed for self‐efficacy in controlling upsetting thoughts (*d* = 1.05), favouring the usual care group. These findings differ from previous studies of single‐component interventions, which have reported limited short‐term effects on behavioural outcomes and carers' responses, with improvements emerging only at later time points (Leng et al. 2020; Saragih et al. 2021). Similarly, a previous study using comparable outcome measures reported no differences in carers' self‐efficacy (Cristancho‐Lacroix et al. 2015). The observed trends in this study may be related to the multi‐component nature of the facilitator‐enabled iSupport program, which integrates education, skill‐building, facilitator support and peer interaction, rather than relying on a single intervention component. These elements may support carers in understanding and responding to behavioural changes, and in applying practical strategies in daily care (Dam et al. 2016). Facilitators in iSupport encouraged family carers to engage with and apply strategies from the iSupport manual through peer support meetings and WhatsApp group messaging. In doing so, they may have acted as catalysts by reinforcing key concepts, prompting reflection and supporting carers to translate the material into practice, although the extent to which this led to sustained understanding and active use of strategies remains unclear. These findings are broadly consistent with prior evidence suggesting that psychoeducational interventions incorporating professional support, problem‐solving and coping strategies can improve carers' capacity to manage behavioural symptoms (Trivedi et al. 2019). Similarly, the inclusion of a facilitator as a single point of contact aligns with recommendations to optimise support for carers managing behavioural and psychological symptoms of dementia (Stephan et al. 2018). However, given the pilot nature of this study and the exploratory analyses, these findings should be interpreted as preliminary and require confirmation in a fully powered trial. Importantly, pilot studies are designed to assess feasibility and trial procedures rather than establish intervention efficacy; therefore, the observed effect sizes should be interpreted as exploratory and hypothesis‐generating.

## 6 Limitations

There are several limitations to this internal pilot study. First, there is potential for selection bias, as carers with greater resources, such as those with sufficient time and capacity to engage with the program and study requirements, may have been more likely to participate. Second, the study included only participants who could read and communicate in English, which limits the generalisability of the findings to the broader, more culturally diverse community. Third, carers may require substantial support during transition periods, such as when PLWD are admitted to permanent residential aged care, a need that was not captured in the present study. Fourth, the impact of the intervention should be interpreted with caution, given the study's pilot nature and limited sample size. Fifth, there are limitations associated with the measurement instruments used. For example, the QOL measures may have been too broad to detect program‐specific effects, as the iSupport intervention does not directly address structural determinants such as physical health, financial circumstances and marital status, which are key components of QOL. Finally, although participants were instructed not to share the iSupport for Dementia Australia version with those in their usual care group, contamination cannot be ruled out, as the WHO version of iSupport for Dementia is freely accessible online.

### 6.1 Recommendations for Further Research

Recruiting carers of PLWD in community settings is challenging; therefore, researchers should allocate sufficient time to achieve target sample sizes. It is also important to consider how the complexity of intervention design may influence both recruitment and retention. Future research should explore additional strategies to better support carers who face barriers such as limited time and competing responsibilities. This may include incorporating screening questions during recruitment to capture factors such as perceived lack of need for the service or the presence of adequate existing support. Furthermore, the use of alternative or complementary measurement tools may help capture a broader range of outcomes, including improvements in depression (e.g., increased engagement in enjoyable activities) and anxiety (e.g., adoption of relaxation techniques), which may better reflect the intervention's effects. Finally, future studies could examine the implementation processes of facilitator‐enabled support models within clinical settings to better understand their feasibility, acceptability and scalability.

### 6.2 Implications for Policy and Practice

Findings from this study indicate that family carers of PLWD were actively engaged in the facilitator‐enabled flexible support program, supporting its feasibility and informing progression to the main trial. The findings suggest that facilitator‐enabled flexible psychoeducational support may be a practical approach for addressing unmet informational, emotional and service‐navigation needs among carers during the post‐diagnostic period. Given nurses' established roles in education, care coordination, psychosocial support and referral to community services, nurses are well positioned to deliver and integrate this model into routine dementia care.

The facilitator‐enabled flexible support model using telephone, virtual peer support groups and WhatsApp may complement existing dementia support services by improving access to timely, person‐centred support, particularly for carers who experience geographical, transport, or time‐related barriers to attending face‐to‐face programs. Regular coordinated contact also provides opportunities for nurses to identify emerging care needs, provide emotional support, strengthen carers' self‐management capabilities and facilitate access to appropriate health and aged‐care services.

These findings are particularly relevant to nurse‐coordinated multidisciplinary memory clinics, community nursing services and other settings that provide remote dementia care and support. The study provides preliminary evidence for incorporating nurse‐facilitated flexible psychoeducational interventions into post‐diagnostic dementia care pathways and highlights the potential role of nurses in expanding access to sustainable, family‐centred dementia support services.

## 7 Conclusion

Caring for PLWD is mentally and physically demanding and stressful for carers. It has a potentially adverse impact on their health and well‐being throughout their caring journeys. This internal pilot RCT provided an opportunity to refine key aspects of the main trial, particularly regarding carer recruitment strategies and intervention delivery formats, whereas also assessing the feasibility and preliminary impact of the iSupport program. The knowledge generated from this study has important implications for the future implementation of facilitator‐enabled interventions. Specifically, it highlights the potential of flexible support models to better meet the diverse needs of carers in the community and to enhance the accessibility and scalability of support services for carers of PLWD.

## Author Contributions

**Julie Ratcliffe:** writing – review and editing, funding acquisition. **Michael Chapman:** funding acquisition, resources, writing – review and editing, supervision, investigation. **Ying Yu:** conceptualization, data curation, formal analysis, writing – original draft, writing – review and editing, project administration, investigation. **Andre Q. Andrade:** writing – review and editing, funding acquisition. **Langduo Chen:** resources, writing – review and editing, funding acquisition, investigation. **Claudia Meyer:** funding acquisition, supervision, validation, writing – review and editing, investigation. **Lily Xiao:** conceptualization, methodology, supervision, funding acquisition, writing – review and editing, validation. **Alison Kitson:** funding acquisition, writing – review and editing. **Sue McKechnie:** resources, writing – review and editing, funding acquisition. **Kai Ping Tan:** writing – review and editing, resources, investigation. **Craig Whitehead:** funding acquisition, writing – review and editing. **Shahid Ullah:** funding acquisition, supervision, writing – review and editing, validation.

## Funding

This study is funded by the Australian Government via the 2020 NHMRC/Medical Research Future Fund and NHMRC/The Dementia Centre for Research Collaboration (DCRC) World‐Class Research Project Grants.

## Disclosure

Statistician on the authorship: Associate Professor Shahid Ullah is a Biostatistician at the College of Medicine and Public Health (CMPH), Flinders University.

## Ethics Statement

Ethics approval was obtained from the Southern Adelaide Clinical Human Research Ethics Committee (no. 2021/HRE00273) and the Bolton Clarke Human Research Ethics Committee (no. 21007).

## Conflicts of Interest

The authors declare no conflicts of interest.

## Supporting information


**File S1:** Carers satisfaction survey.

**File S2:** Intervention effectiveness outcome measure instrument.

**File S3:** Semi‐structured interview guide (site leaders, facilitators and family carers).

**File S4:** Good Reporting of A Mixed Methods Study (GRAMMS) checklist.

**File S5:** CONSORT 2010 checklist of information to include when reporting a pilot or feasibility trial.*

## Data Availability

De‐identified data related to the outcome measures published from the study will be shared on request.
