# Supplementary material for: Exploring a Facilitator‐Enabled Flexible iSupport for Dementia Program: An Internal Pilot Mixed‐Methods Study
Source: Nurs Open. 2026 Aug 23;13(8):e70774. doi: 10.1002/nop2.70774 (PMC13501002; doi:10.1002/nop2.70774)
Supplement: Supplementary file 1 [file NOP2-13-e70774-s002.pdf]

Site code _____ personal code_____ Month___________

How many units in iSupport manual have you completed?

____

If you have not completed any units in the iSupport manual, can you please explain the reason?

____

____

Carer in the iSupport group satisfaction survey

Items	1<br>Strongly disagree	2<br>Disagree	3<br>Neither agree nor disagree	4<br>Agree	5<br>Strongly a gree	6<br>N/A
1.My facilitator motivates me to learn from the online iSupport program.						
2.My facilitator helped me identify learning units of the iSupport that are relevant to my situation.						
3.My facilitator helped me identify healthcare/social care services my care recipient and/or myself need.						
4.My facilitator motivates me to participate in the care support group meeting.						
5.I am satisfied with the carer support group meeting.						
6.My facilitator motivates me to interact with other carers on a weekly basis.						
7.I am satisfied with the weekly carer interactions via chat and text messages.						
8.My facilitator respond to my request for support on a timely manner.						
9.I am satisfied with the individualised support my facilitator provided to me.						
10.Overall, I am satisfied with the support my facilitator provided to me.						

Other comments:
