# Supplementary material for: Exploring a Facilitator‐Enabled Flexible iSupport for Dementia Program: An Internal Pilot Mixed‐Methods Study
Source: Nurs Open. 2026 Aug 23;13(8):e70774. doi: 10.1002/nop2.70774 (PMC13501002; doi:10.1002/nop2.70774)
Supplement: Supplementary file 2 [file NOP2-13-e70774-s004.pdf]

Semi-structured interview guide
(site leaders, facilitators and family carers)

Title: Creating 'partnerships in iSupport program' to optimise carers' impact on dementia care

Part 1: Demographic information

You are a:

a. Site leaders
b. Facilitator
c. Family carer

1. For Facilitator:
    a. Gender
    b. Age
    c. What is the highest level of education you achieved?
    d. What is your current employment role apart from iSupport facilitator? (i.e., RN, Physio)
    e. How long have you worked in your current role?
    f. How long have you been employed in your current organization?
2. For family carers:
    Collected in the survey

Part 2: Explore your experiences in iSupport program for dementia.

Sime-structure interview questions with site-specific leaders and facilitators

1. How do you think the 'Partnership in iSupport program' has influenced carers in the care of their loved ones while reducing their stress? Please discuss and give some examples?
2. How have the facilitator's role and activities helped carers improve their caregiving performance? Please discuss which aspects of carers' performance (i.e. knowledge, skills, positive thoughts, navigating and access care services) have you observed and give some examples?
3. What positive experiences have you had in the program? Please provide some examples.
4. What difficulties have you experienced during the program? How have you managed these difficulties? Please give some examples.
5. What kind of support have you received from your organisation and how has the support helped you in the program?
6. What kind of support have you received from management and how has the support helped you?
7. What kind of support have you received from staff/peers and how has the support helped you?
8. What could be done differently to improve the implementation of the program?
9. What strategies will enable your organization to embed and sustain the program after this clinical trial?
10. What further comments do you have?

Sime-structure interview questions with carers

1. How do you think the 'Partnership in iSupport program' has influenced you in the care of your loved ones living with dementia while reducing your stress? Please discuss and give some examples?
2. How have the support from the facilitator helped you improve your caregiving performance? Please discuss which aspects of your performance (i.e. knowledge, skills, positive thoughts, navigating and access care services) have you improved and give some examples?
3. How have the carer support meetings helped you improve your caregiving performance? Please discuss which aspects of your performance (i.e. knowledge, skills, positive thoughts, navigating and access care services) have you improved and give some examples?

4. How have the weekly text message interactions with other carers helped you improve your caregiving performance? Please discuss which aspects of your performance (i.e. knowledge, skills, positive thoughts, navigating and access care services) have you improved and give some examples?
5. What positive experiences have you had in the program? Please provide some examples.
6. What negative experiences have you had in the program? Please provide some examples.
7. What difficulties have you experienced during the program? How have you managed these difficulties? Please give some examples.
8. What could be done differently to improve the implementation of the program?
9. What strategies will enable the care service providers to embed and sustain the iSupport program after this clinical trial?
10. What further comments do you have?
