# Supplementary material for: Exploring a Facilitator‐Enabled Flexible iSupport for Dementia Program: An Internal Pilot Mixed‐Methods Study
Source: Nurs Open. 2026 Aug 23;13(8):e70774. doi: 10.1002/nop2.70774 (PMC13501002; doi:10.1002/nop2.70774)
Supplement: Supplementary file 3 [file NOP2-13-e70774-s003.docx]

Supplementary File 2: Intervention effectiveness outcome measure instrument

| **Survey** | **Item** | **Survey details** | | **Scoring** | **Score direction** | **Validity** | **Reliability** |
| --- | --- | --- | --- | --- | --- | --- | --- |
| QOL: 12-item Short-Form Health Survey version 2  (SF-12 Health Survey v2) (Ware et al., 1996). | 12 | Physical health-related domains | carer’s general health, physical function, and pain | 0-100 | Higher=better | 0.91-0.92 | 0.76-0.89 |
|  |  | Mental health-related domains: | Vitality, social functioning, role emotional and mental health. |  |  |  |  |
| QoL in Alzheimer’s Disease (QoL-AD)-Proxy-Family Version (Logsdon et al., 1999) | 13 | Physical condition, changed behaviours, memory, mood, relationship, financial situation, ability to participate in meaningful activity and the overall QOL | | 13-52 | Higher=better | 0.84-0.86 | 0.76-0.92 |
| Caregiving self-efficacy scale (Steffen et al., 2002) | 15 | Self-efficacy for obtaining respite (seeking help from family and friends | | 0-100 | Higher=better | 0.80 | 0.70 |
|  |  | responding to atypical patient behaviours | |  |  |  |  |
|  |  | controlling upsetting thoughts about caregiving | |  |  |  |  |
| The Carers of Older People in Europe Index-Quality of Social Support (QOS) (McKee et al., 2003) | 5 | quality of social support (from friends, neighbours, family, service providers) | | 0-20 | Higher=better | 0.76 | 0.80 |
| Revised Memory and Behaviour Problem Checklist (RMBPC) (Teri et al., 1992) | 24 | Frequency of the behaviour | | 0-96 | Lower=better | good | 0.84-0.90 |
|  |  | Carer’s reaction | |  |  |  |  |
