# Supplementary material for: Exploring a Facilitator‐Enabled Flexible iSupport for Dementia Program: An Internal Pilot Mixed‐Methods Study
Source: Nurs Open. 2026 Aug 23;13(8):e70774. doi: 10.1002/nop2.70774 (PMC13501002; doi:10.1002/nop2.70774)
Supplement: Supplementary file 4 [file NOP2-13-e70774-s005.docx]

**Good Reporting of A Mixed Methods Study (GRAMMS) checklist**

| **Guideline** | **Section: page** |
| --- | --- |
| Describe the justification for using a mixed methods approach to the research question | Methods- 3.1 study design, page 7 line 12-19 |
| Describe the design in terms of the purpose, priority and sequence of methods | Methods  Page 7- 12, Figure 1 |
| Describe each method in terms of sampling, data collection and analysis | Methods  3.2 Setting, participants and ethics consideration Page 7  3.5 Sample size: Page 10  3.8 Data collection: Page 11-12  3.9 Data analysis: Page 12-13 |
| Describe where integration has occurred, how it has occurred and who has participated in it | Method  3.1 Study design Page 7  3.9.3 Mixed methods data analysis, interpretation, and integration: Page 13 |
| Describe any limitation of one method associated with the present of the other method | Limitations Page 23 |
| Describe any insights gained from mixing or integrating methods | Discussion Page 18-23 |

O'Cathain, A., Murphy, E., & Nicholl, J. (2008). The quality of mixed methods studies in health services research. *JOURNAL OF HEALTH SERVICES RESEARCH & POLICY*, *13*(2), 92-98. https://doi.org/10.1258/jhsrp.2007.007074
